# Improving central line-associated bloodstream infection prevention practices in oncology clinic patients: mobile-app based surveillance & response

**DOI:** 10.1017/ice.2025.16

**Published:** 2025-05

**Authors:** Hiroki Saito, Shereen Nourollahi, Mohamad N. Alsharif, Bardia Bahadori, Tom Tjoa, Amarah Mauricio, Jessica Bethlahmy, Justin Chang, Syma Rashid, Edward L. Nelson, Richard A. Van Etten, Linda Armendariz, Victor Torres, Sandra Masson, Marlene Esteves, Raheeb Saavedra, Raveena D. Singh, Shruti K. Gohil

**Affiliations:** 1 Division of Infectious Diseases, University of California Irvine, School of Medicine, Irvine, CA, USA; 2 St. Marianna University School of Medicine Yokohama Seibu Hospital, Yokohama, JAPAN; 3 Department of Medicine, Division of Oncology, UC Irvine, School of Medicine, Irvine, CA, USA; 4 Irvine Health, Chao Cancer Center, University of California, Irvine, CA, USA; 5 Epidemiology & Infection Prevention, UC Irvine Health, Irvine, CA, USA

## Abstract

**Objectives:**

To evaluate the impact of a mobile-app-based central line-associated bloodstream infection (CLABSI) prevention program in oncology clinic patients with peripherally inserted central catheters (PICCs).

**Design:**

Pre-post prospective cohort study with baseline (July 2015–December 2016), phase-in (January 2017–April 2017), and intervention (May 2017–November 2018). Generalized linear mixed models compared intervention with baseline frequency of localized inflammation/infection and dressing peeling. Cox proportional hazards models compared days-to-removal of lines with localized inflammation/infection. Chi-square test compared bacteremia rates before and after intervention.

**Setting:**

Oncology clinic at a large medical center.

**Patients:**

Oncology clinic adult patients with PICCs.

**Intervention:**

CLABSI prevention program consisting of an actionable scoring system for identifying insertion site infection/inflammation coupled with a mobile-app enabling photo-assessments and automated physician alerting for remote response.

**Results:**

We completed 5,343 assessments of 569 PICCs in 401 patients (baseline: 2,924 assessments, 300 PICCs, 216 patients; intervention: 2,419 assessments, 269 PICCs, 185 patients). The intervention was associated with a 92% lower likelihood of having a dressing with peeling (OR 0.08, 95%CI 0.04-0.17, *P* < 0.001), 53% lower local inflammation/infection (OR 0.47, 95%CI 0.27-0.84, *P* < 0.011), and 24% (non-significant) lower CLABSI rates (*P* = .63). Physician mobile-app alerting and response enabled 80% lower risk of lines remaining in place after inflammation/infection was identified (HR 0.20, 95%CI:0.14-0.30, *P* < 0.001) and 85% faster removal of infected lines from mean (SD) 11.1 (9.7) to 1.7 (2.4) days.

**Conclusions:**

A mobile-app-based CLABSI prevention program decreased frequency of inflamed/infected central line insertion sites and increased speed of removal when inflammation/infection was found.

## Background

Increasingly complex medical care is being delivered in post-discharge settings. This includes millions of patients with cancer who are discharged from hospitals with peripherally inserted central catheters (PICCs) that are largely managed by patients at home and outpatient clinic staff.^1–4^ These patients are often immunocompromised, placing them at exceptionally high risk for central-line-associated bloodstream infections (CLABSIs), which are associated with extensive morbidity, mortality, readmissions, and substantial excess healthcare costs.^5–8^ Though significant strides have been made in preventing CLABSIs in hospital settings, strategies to assure high-quality central line maintenance care processes are poorly defined in outpatient settings.^4,9–12^

Reliable and proactive CLABSI prevention practices that focus on monitoring, maintenance, and timely response to high-risk lines are needed for patients at home with peripherally inserted central catheter (PICC) lines. We evaluated the impact of an outpatient CLABSI prevention bundle known as the SAFER (Standardizing Assessment For Effective Response) Lines program that allows remote, mobile-app-based central line monitoring and physician response when high-risk lines are identified.

## Methods

We conducted a pre-post prospective cohort study of the SAFER Lines program in adults (age ≥ 18) with PICCs placed in patients undergoing chemotherapy at an outpatient oncology clinic at a large academic medical center in Orange County, California. The study included a baseline observation period (July 2015 – December 2016), phase-in period (January 2017 – April 2017), and intervention period (May 2017– November 2018). The SAFER Lines bundle was implemented as a quality improvement protocol and approved by the institutional review board of the University of California, Irvine as a minimal-risk study with waiver of informed consent.

### Baseline activities

**Figure 1.**
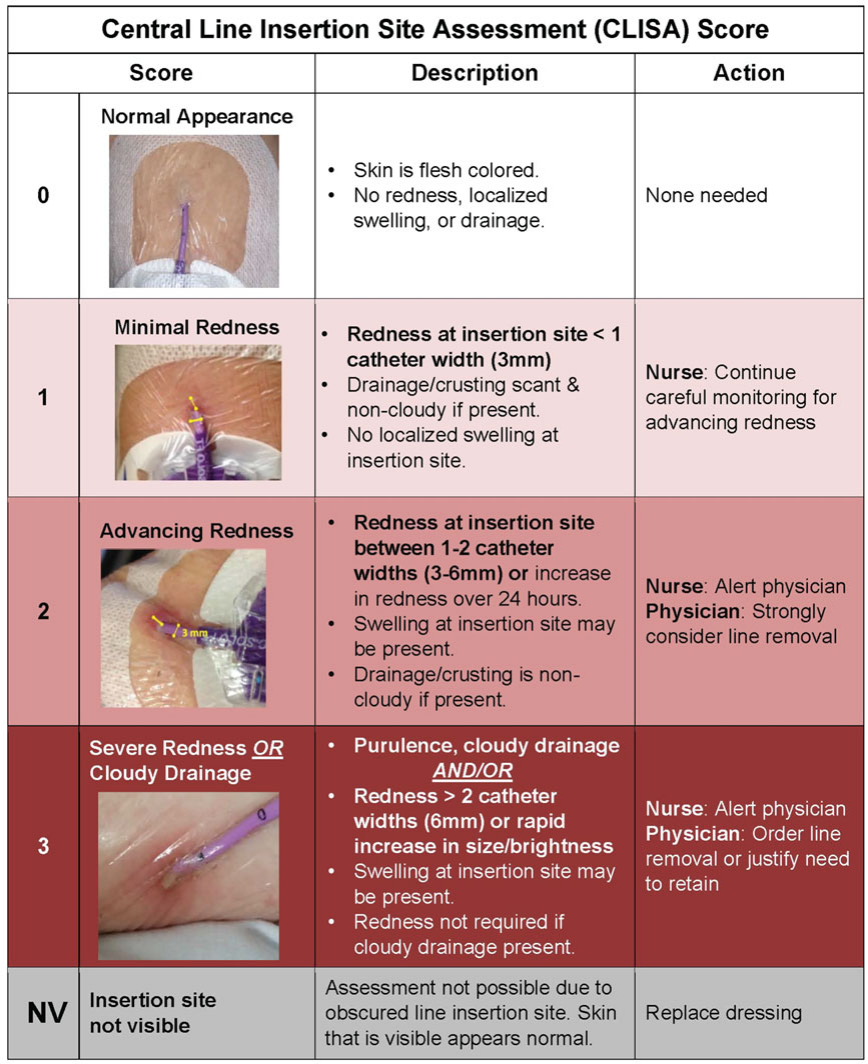
Central Line Insertion Site Assessment (CLISA) Score provides a framework for assessing and interpreting the presence of localized inflammation or infection at the skin surrounding the insertion site. The width of the catheter size is used to estimate the extent and grade of erythema. Each score is linked with recommended clinician actions, with an expectation to remove central lines with high risk of progression to bloodstream infections (score of 2 or 3).

In the baseline period, research staff conducted daily (Monday-Friday) photo-assessments of PICC line insertion sites and dressings and recorded the presence of localized inflammation or infection using the Central Line Insertion Site Assessment (CLISA) scoring system (Figure 1), completing observations without intervention.

### Intervention activities

During the phase-in period, the SAFER Lines program was implemented. It consisted of 3 elements:

(1) CLISA score (Figure 1) with patient, nurse, and physician education on how to use the score and its expected actions.^13^ Continuation of assessments for other signs of infection including fever, vital signs, and non-visual elements of the skin exam (e.g., palpation for tenderness and warmth) was also encouraged.

(2) Use of the SAFER Lines mobile-app by nurses, designed to facilitate daily photo-assessments of central lines for early identification of insertion site inflammation or infection and within-app response to high-risk lines to prevent progression to CLABSI (Supplemental Figure 1A-B).

(3) Dressing maintenance education for nurses (placement, appearance, frequency of changes, and scrub-the-hub practices for line access). This consisted of an in-person verbal presentation and written education.

Briefly, the CLISA score provides a framework for categorizing insertion site inflammation, standardizing erythema by catheter-width (3mm) as a reference and CLISA score is tied to a recommended response. CLISA 1 indicates erythema < 3mm radius and warrants watchful waiting; CLISA 2 indicates 3-6mm of erythema indicating localized inflammation with potential for progression, warranting strong consideration for removal, and CLISA 3 indicates > 6mm of erythema or presence of any pus regardless of erythema, warranting urgent removal.

The SAFER Lines mobile-app enabled (a) daily photo-documentation of insertion sites and CLISA score entry by nurses, (b) automated physicians alerts when high-risk lines were identified (CLISA score 2 or 3, indicating localized inflammation or infection), (c) remote physician examination of current and past insertion site photos, and (d) within-app physician response and ordering for timely response (e.g., continue monitoring, remove PICC, place peripheral intravenous catheter), Supplemental Figure 1A-B. The SAFER Lines mobile-app is a HIPAA-compliant web-based application whereby patient data are encrypted and stored on a centrally secured, firewall-protected webserver at the University of California, Irvine. The oncology clinic was given an iPad-Mini for each nursing station; nurses and physicians were trained and individually enrolled with secured username/password logons. Physicians downloaded the SAFER Lines mobile-app (compatible with both Apple and Android operating systems) onto their own mobile devices.

During the phase-in and intervention periods, nurses used the mobile-app to monitor insertion sites of all patients with central lines in the oncology clinic, entering the CLISA score and a photograph of the insertion site and dressings daily. Physicians received automated alerts for CLISA scores of 2 or 3 via text and email, viewed photo-assessments, and responded to messages within the mobile-app to order removal, replacement, or monitoring of the line. The app allows nurses to receive physician orders and review whether the provider viewed alerts/messages. If CLISA scores of 2 or 3 were not viewed by physicians within 4 hours, nurses were instructed to send a repeat message through the mobile-app and page/call physicians directly.

Research staff remotely monitored nursing photo-assessments and physician responses in real-time through a secured webserver where photographs and mobile-app entries were captured. Research staff independently assigned CLISA scores; any discrepancies with nursing scores prompted escalation to physician investigators for score verification and direct nursing feedback and education as needed. Research staff went to clinics thrice weekly to encourage compliance with CLISA scores and mobile-app photo-assessments.

### Data collection

During baseline, research staff collected daily photo-assessments, assigned CLISA scores, dressing integrity (peeling on one or more sides), and nursing or physician chart documentation of line care or insertion site appearance. Line insertion dates were recorded per chart documentation (physician, nursing, or procedure notes). Line removal dates and times were recorded per chart documentation and confirmed verbally with nurses.

During intervention, nurses completed daily photo-assessments using the SAFER Lines mobile-app as described above, entering CLISA scores, dressing integrity, line insertion and removal dates/times; all dates were confirmed retrospectively by research staff through chart review, with verbal verification with nursing staff as needed. All analyses were completed using CLISA scores as assigned by research staff to minimize interobserver variability.

Finally, research staff conducted chart reviews for all patients to obtain bacteremia events up to 90 days after the last PICC assessment and evaluated for CLABSI using Centers for Disease Control and Prevention’s National Healthcare Safety Network criteria and attributed to the PICC line if alternative primary source for bacteremia was not identified.^14^

### Outcomes

The following outcomes were evaluated: 1) CLISA 2, CLISA 3, and the composite of CLISA 2 or 3 as indicators of localized inflammation or infection; 2) days-to-line removal from first CLISA 2 or 3 (composite) and the subsets of CLISA 2 and 3; 3) presence of any peeling on dressings; 4) CLABSI.

### Statistical analysis

Patients were described by age, sex, history of prior PICC line, malignancy type (solid tumor versus hematologic malignancy). PICC line characteristics and dressing integrity were evaluated per line and across assessments. Data from phase-in were not included in any analyses. Lines were described by frequency, dwell time (summed days from insertion to removal), maximum CLISA scores recorded during each line’s dwell time (each line was assigned into a mutually exclusive category), and days-to-line removal (summed days from first maximum CLISA score to date of line removal). Lines without an available removal date were not included in dwell time or days-to-line removal calculations. Photo-assessments where line insertion sites were not visible (presence of gauze or blood or photos that were too dark or blurry) were not included in analysis. We additionally assessed the proportion of lines with dressings that had any peeling. Chi-squared tests were used to compare baseline versus intervention proportions of lines with abnormal CLISA scores and peeling dressings. T-tests were used to compare mean dwell time and days-to-line removal after an abnormal CLISA score of 2 or 3.

Generalized linear mixed-effects models evaluated the effect of the SAFER Lines program on the following outcomes, accounting for clustering by patient: (1) CLISA 2, CLISA 3, and the composite of CLISA 2 or 3 in separate models with independent variables of study period, age (years), sex, line dwell time (days), dressing peeling on one or more sides, history of prior PICC line, and malignancy type; (2) proportion of lines with peeling dressings adjusting for age, gender, line dwell time, malignancy type, and history of prior line. Kaplan-Meier curves were generated for the duration that lines were retained with an abnormal CLISA score; Cox proportional hazards models (adjusted for age, gender, history of prior line, and malignancy type) were used to evaluate the effect of the intervention on these outcomes while accounting for clustering at the patient-level. All analyses were completed using SAS version 9.4 (Cary, NC).

## Results

Table 1 summarizes patient characteristics. There were a total of 5,343 assessments of 569 lines in 401 patients across the study periods, with 2,924 assessments of 300 PICCs in 216 patients during baseline and 2,419 assessments of 269 PICCs in 185 patients during intervention.

**Table 1.** Characteristics of participating oncology clinic patients with peripherally inserted central catheters

	Baseline N (%) 15 months	Intervention N (%) 19 months
Patient characteristics^a^		
Number of patients with central lines (N)	216	185
Age in years (mean, SD)	58 (17)	53 (17)
18-45	56 (26)	68 (37)
46-55	37 (17)	26 (14)
56-65	44 (20)	44 (24)
66-75	52 (24)	32 (17)
>75	27 (13)	15 (8)
Male sex	116 (58)	115 (64)
Prior central line	48 (22)	56 (30)
Hematologic malignancy	109 (50)	132 (71)
Solid Tumor	90 (42)	49 (27)
Total Number of PICC lines	300	269
Assessments completed	2,924	2,419

a Patients with peripherally inserted central catheter (PICC) line in place.

### Impact of SAFER lines program on central line maintenance practices

While the SAFER Lines program focused primarily on monitoring insertion sites, identifying sites with CLISA 2 or 3, and responding with line removal in a timely manner, we observed improvements in maintenance activities. Comparing baseline to intervention, there was an 80.4% decrease in lines with peeling dressings, from 30.7% (92/300) to 6.0% (15/269) and in adjusted analyses, the SAFER Lines program was associated with a 92% lower likelihood of having a dressing with peeling (OR 0.08, 95% CI 0.04-0.17, *P* < 0.001). Daily nursing documentation of dressing and insertion site appearance improved from < 1% during baseline to > 98% in the last month of the study period.

### Impact of SAFER lines program on risk of developing CLISA 2 or 3

Among lines with at least one assessment of a visible insertion site, the proportion with localized infection or inflammation (CLISA 2 or 3) decreased from 40.7% (122/300) during baseline to 26.0% (70/269) during intervention (36% reduction, *P* < 0.001), Table 2. Concomitantly, CLISA 0 or 1 increased from 50.0% (150/300) to 69.1% (186/269) from baseline to intervention, respectively. On adjusted analyses (Table 3), the SAFER Lines program was associated with 53% lower odds of developing CLISA 2 or 3, OR = 0.47 (95%CI 0.27-0.84, *P* < 0.011) and a 63% lower odds of developing a CLISA 3, OR = 0.37 (95%CI 0.16-0.81, *P* < 0.014). Notably, peeling dressings were significantly associated with an increased risk of developing a CLISA 2 or 3, adjusted OR = 2.55 (95%CI 1.31-4.97, *P* = 0.006), (Table 3).

**Table 2.** Dressing integrity, presence of insertion site inflammation or infection, and days-to-line removal during baseline and intervention periods

	Baseline N (%) 15 months	Intervention N (%) 19 months	*P*-value (Chi Square)
PICC lines with visible insertion sites^a^	272	256	
PICC line dwell time (mean, SD)	90.9 (86.7)	89.9 (92.1)	0.894
Dwell time - visible lines only (mean, SD)	92.8 (88.8)	91.6 (93.4)	0.879
Dressings peeling on ≥ 1 Side	92 (30.7)	16 (5.9)	<0.001
Peeling 1 side only	61 (25.3)	15 (5.6)	<0.001
Peeling 2 or more sides	31 (10.3)	1 (0.4)	<0.001
No Peeling	208 (69.3)	240 (93.8)	<0.001
Maximum CLISA score^b^			<0.001
CLISA 0	56 (18.7)	73 (27.1)	
CLISA 1	94 (31.3)	113 (42.0)	
CLISA 2	71 (23.7)	41 (15.2)	
CLISA 3	51 (17.0)	29 (10.8)	
CLISA 0 or 1 (composite)	150 (50.0)	186 (69.1)	<0.001
CLISA 2 or 3 (composite)	122 (40.7)	70 (26.0)	<0.001
Days-to-line removal^c^			
From first CLISA 2 (mean, SD)	18.6 (9.7)	6.9 (3.7)	<0.001
From first CLISA 3 (mean, SD)	11.1 (9.8)	1.7 (2.4)	<0.001
From first CLISA 2 or 3 (mean, SD)	16.0 (10.3)	4.7 (4.1)	<0.001
All visible assessments			<0.001
CLISA 0	1,526 (52.2)	1,541 (63.7)	
CLISA 1	596 (20.4)	695 (28.7)	
CLISA 2	318 (10.9)	79 (3.3)	
CLISA 3	107 (3.7)	32 (1.3)	

a Data presented are for lines with one or more insertion site assessments that were visible. During baseline and intervention periods the number of lines that could not be assessed due to insertion sites being covered by blood or gauze were 28 and 13 lines respectively.

b Mutually exclusive categories with each line assigned according to maximum CLISA (central line insertion site assessment) Score through line duration.

c Calculated using date of first maximum CLISA score to date of line removal; for lines without a known removal date, the last day of line assessment was used.

**Table 3.** Multivariable model: impact of the SAFER lines CLABSI prevention bundle on proportion of lines with localized inflammation or infection^a^ ^,^ ^b^

Variables	Local inflammation or infection at insertion site					
	CLISA Score 2 or 3		CLISA Score 2		CLISA Score 3	
	Odds Ratio (CI)	*P*-value	Odds Ratio (CI)	*P*-value	Odds Ratio (CI)	*P*-value
SAFER lines intervention	0.47 (0.27-0.84)	0.011	0.46 (0.26-0.83)	0.011	0.37 (0.16-0.81)	0.014
Age		0.056		0.027		0.317
46-55	2.10 (0.94-4.69)		1.82 (0.79-4.19)		2.39 (0.90-6.36)	
56-65	1.95 (0.93-4.08)		2.04 (0.93-4.45)		1.29 (0.50-3.30)	
66-75	2.92 (1.37-6.21)		3.77 (1.68-8.45)		1.14 (0.45-2.93)	
>75	1.10 (0.43-2.79)		1.30 (0.49-3.50)		0.59 (0.16-2.20)	
Male sex	0.70 (0.42-1.18)	0.178	0.75 (0.43-1.28)	0.283	0.60 (0.31-1.16)	0.126
Hematologic malignancy	1.62 (0.90-2.93)	0.108	1.39 (0.76-2.55)	0.279	2.20 (0.98-4.98)	0.057
History of prior central line	1.77 (1.03-3.04)	0.040	1.43 (0.82-2.49)	0.206	2.15 (1.06-4.35)	0.034
Dressing peeling on ≥ 1 side	2.55 (1.31-4.97)	0.006	2.51 (1.28-4.94)	0.008	1.04 (0.46-2.35)	0.921
PICC line dwell time, mean (days)	0.99 (0.99-0.99)	<0.001	0.99 (0.99-1.00)	<0.001	0.98 (0.97-0.99)	<0.001

a Results are based on generalized linear mixed-effects models done at the PICC line level, accounting for clustering within patients.

b SAFER, Standardizing Assessments For Effective Response; CLABSI, central line-associated bloodstream infection. Referents for each categorial independent variable were as follows: SAFER Lines intervention referent = baseline period, age = 18-45 years, Sex referent = female, hematologic malignancy referent = solid tumor, history of prior line referent = no prior line, dressing peeling referent = no peeling during duration of line.

### Impact of SAFER lines program on days-to-line removal after CLISA 2 or 3

Removal of lines after CLISA 2 or 3 occurred 71% faster, from a mean of 16.0 (SD = 10.3) days during baseline to 4.7 (4.1) days after intervention (*P* < 0.001), Table 2. In the subset of lines with CLISA 3, removal was 85% faster, from 11.1 (9.8) days to 1.7 (2.4) days during the baseline versus intervention periods, respectively.

**Figure 2.**
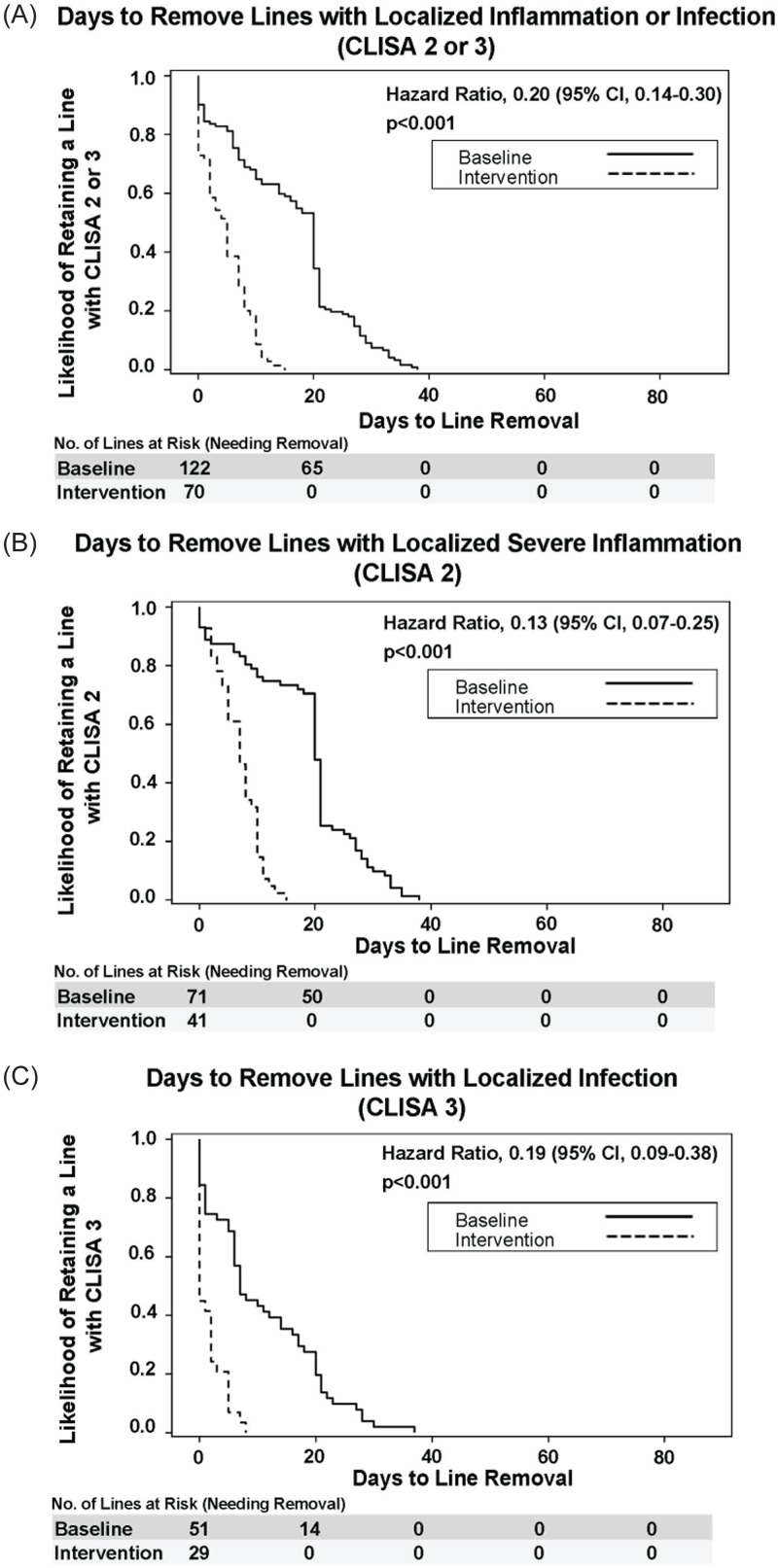
Probability of Removal of Lines Identified with Inflammation or Infection During the Baseline versus Intervention Periods. A–C: Kaplan-Meier curves for estimated probability of line removal when localized inflammation or infection are identified according to (2A) CLISA (Central Line Insertion Site Assessment) scores of 2 or 3, composite of localized inflammation or infection; (2B) CLISA score 2 indicating progressive localized inflammation (2C) CLISA score 3 indicating severe inflammation or infection (severe erythema or purulence). Cox proportional hazards modeling was used to evaluate days-to-removal for baseline and intervention periods, adjusting for age, gender, history of prior line, and malignancy type.

The SAFER Lines program was associated with 80% lower risk of a line remaining in place once CLISA 2 or 3 was identified [HR 0.20 (95%CI 0.14-0.30), *P* < 0.001], with similar findings for the subsets CLISA 2 and 3 individually. Kaplan-Meier curves and proportional hazards model results for days-to-line removal upon first identification of CLISA 2 or 3 are shown for baseline and intervention periods in Figures 2A-C.

### Bacteremia

Across the study period, a total of 15 patients developed a CLABSI (0.25/1,000 line-days). The proportion of patients who developed bacteremia and met CDC criteria for CLABSI decreased by 24% from 4.2% (9/216) to 3.2% (6/185) during the intervention and baseline periods however this was not statistically significant (*P* = 0.627).

## Discussion

Patients with cancer receiving intravenous chemotherapy are at high risk for bacteremia and hospital admissions.^8,15^ Barriers to delivering hospital-quality CLABSI prevention practices in outpatient settings include lack of daily nursing or physician monitoring, high physician and nurse-to-patient ratios, lack of standardized infection prevention processes and training within clinics, and reliance on patients to maintain central line care processes at home.^11,12,16–19^ When high-risk lines are identified, preventive responses can be delayed due to challenges in timely communication and care coordination between nurses and physicians.^19,20^ We found that mobile-app-based photo-monitoring of central line insertion sites for within-app physician responses decreased the risk of local inflammation or infection (CLISA 2 or 3) by 53% and lowered the risk of retention when CLISA 2 or 3 are identified by 80%.

The SAFER Lines CLABSI prevention bundle was associated with multiple improvements in line care. First, dressing maintenance improved, including an 80% improvement in fully intact dressings and near-perfect adherence with documenting visual assessment. This decrease is noteworthy given that the mobile-app did not include triggered actions for dressing integrity. While we provided dressing education, our experience was that mobile-app photographs increased the frequency and detail with which nurses evaluated dressings, resulting in improved maintenance. Importantly, we found that peeling dressings are associated with a 2.6-fold higher risk of insertion site inflammation or infection (CLISA 2 or 3), highlighting the importance of maintaining dressing integrity in preventing localized infection in outpatient settings. Second, the prevalence of PICC insertion site inflammation or infection was reduced by 41% and removal of CLISA 2 or 3 lines occurred 4-times faster, with the subset of CLISA 3 lines removed within less than 2 days.

The success of the SAFER Lines program was likely due to its ability address several implementation barriers to infection prevention in outpatient settings. By providing a common language and standardized metrics across care providers to identify signs of localized infection with associated expectations for action, the CLISA score facilitated early identification of high-risk lines and timely response.^13^ Additionally, physician auto-alerting for CLISA 2 or 3 allowed remote examinations of photos with assessment of changes over time through serial photos, which provided physicians with all necessary information for clinical decision-making, allowing them to place orders within the app to promote timely action.

We also observed a 24% lower CLABSI rate after the SAFER Lines intervention which was not statistically significant, likely due to an insufficient sample size and event rate to effectively evaluate this outcome. Nevertheless, since extraluminal introduction of pathogens is a known pathway for the development of CLABSI and our intervention was designed to target local insertion site inflammation and infection, the reduced CLABSI rates after intervention provide a meaningful signal for further study in larger cohorts.^21,22^

Our intervention is an example of a successful telemedicine approach to facilitate infection prevention in outpatient settings. The mobile-app allowed meaningful communication between doctors and nurses about non-emergent findings and encouraged proactive measures for patient safety that might otherwise go undiscussed. Nurses in clinics and patients at home often await regularly planned physician visits to bring non-urgent issues to physician attention unless they require immediate diagnostics or treatments. In the case of central line-associated events, the usual trigger for a nurse to contact a treating physician would be fever, overt evidence of infection, and acute pain at the line. By providing a way to alert physicians with high-yield information about a high-risk line through a mobile-app, nurses could obtain physician attention for preventive action before severe symptoms arose.

Successful implementation of our intervention would need to address some challenges. Acceptance and facility with digital processes was variable and initially lower among older physicians. Upon education that alerts were restricted to only symptomatic lines and that mobile-app functionality included the ability to view photos and order treatment within the app, physicians quickly recognized the value of our approach. This highlights key elements of mobile-app design that can increase acceptance: (1) programming alerts to trigger only when pertinent for clinical response, (2) inclusion of actionable information for clinical decision-making (in our case, a photo of a concerning skin finding), and (3) within-app ordering functionality to facilitate timely response. Additionally, to address medical-legal concerns that messages sent by nurses might not be received by physicians or vice-versa, nursing and physician user interfaces displayed whether messages had been received and read, with timestamps.

Our study has several important limitations. First, CLISA evaluation was limited to only insertion sites that were visible, which impacted our sample size. Despite these sample size limitations, we saw dramatic and statistically significant improvements in CLISA scores and response to high-risk lines. Second, we are unable to differentiate the relative impact of the mobile-app versus education in our bundled intervention, highlighting an area of future study. Third, our quasi-experimental design precludes determination of causality. Fourth, data are limited to study participants, we are unable to provide population-level CLABSI rates. Fifth, the mobile-app did not have white balance or photo-normalization capability, which could have introduced variation in photo quality; our analysis was limited to assessable photos. Finally, in patients with darker skin tones, erythema can be difficult to visualize during exam or in photos, resulting in higher risk for unrecognized skin/soft tissue infections and late presentation with sepsis, higher morbidity, and mortality.^23–28^ Solutions to detect infection earlier in darker pigmented patients are urgently needed. In our experience, photo-assessments captured purulence (CLISA 3) and dressing maintenance targets (peeling, soiling) in residents with darker skin tones.

The SAFER Lines program successfully improved dressing quality, reduced localized inflammation and infection at PICC insertion sites, and facilitated rapid removal of lines at high risk for progression to CLABSI in outpatient oncology clinic patients. This work provides evidence supporting a new strategy for CLABSI prevention that is tailored to address operational barriers in the outpatient setting and highlights the gains that can be achieved through photo assessments and mobile-app-based approaches.

## Supporting information

Saito et al. supplementary material Saito et al. supplementary material

## Supplementary material

The supplementary material for this article can be found at https://doi.org/10.1017/ice.2025.16
