# Supplementary material for: Improving central line-associated bloodstream infection prevention practices in oncology clinic patients: mobile-app based surveillance & response
Source: Infect Control Hosp Epidemiol. 2025 Apr 10;46(5):465–71. doi: 10.1017/ice.2025.16 (PMC12034452; doi:10.1017/ice.2025.16)
Supplement: Saito et al. supplementary material [file S0899823X25000169sup001.docx]

**Supplemental Tables and Figures**

**Supplemental Figure 1**

**
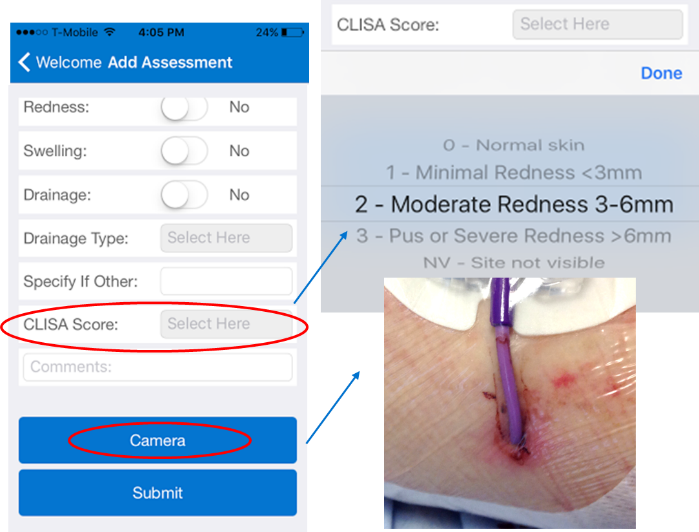

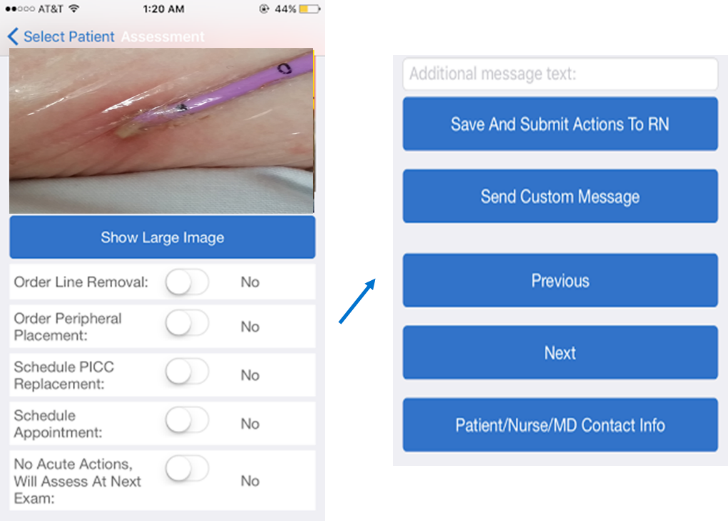
**

**B. Physician Response Within Mobile-App**

**A. Mobile-App Nursing Photo-Assessments**

**Supplemental Figure 1A-B: The Standardized Assessments For Effective Response (SAFER) Lines Mobile App**: The SAFER Lines mobile app was designed to allow remote clinician monitoring of central line insertion sites and response when high risk central lines are found. **(A)** Nurses use mobile-app to take photo-assessments and record the central line insertion site assessment (CLISA) score. **(B)** Physicians receive alerts when a high risk CLISA score of 2 or 3 are identified, enabling remote examination of the insertion and within-app ordering of appropriate actions. All data are stored on a secured web-based platform (not on the mobile device).
